# A cross‐sectional assessment of injection of “salts” and HIV transmission‐related behaviours among a cohort of people who inject drugs in Kyrgyzstan

**DOI:** 10.1002/jia2.26247

**Published:** 2024-07-08

**Authors:** Rebecca Kennedy, Zachary Bouck, Dan Werb, Ainura Kurmanalieva, Anna Blyum, Natalya Shumskaya, Thomas L. Patterson, Javier A. Cepeda, Laramie R. Smith

**Affiliations:** ^1^ School of Medicine University of California San Diego La Jolla California USA; ^2^ Centre on Drug Policy Evaluation Unity Health Toronto Toronto Ontario Canada; ^3^ AIDS Foundation‐East West in the Kyrgyz Republic Bishkek Kyrgyzstan; ^4^ Department of Psychiatry University of California San Diego La Jolla California USA; ^5^ Bloomberg School of Public Health Johns Hopkins University Baltimore Maryland USA

**Keywords:** drug use, PrEP, risk factors, HIV prevention, key and vulnerable populations, public health

## Abstract

**Introduction:**

Despite the increasing availability of new psychoactive substances (hereafter referred to as “salts”) in Eastern Europe and Central Asia, there is a dearth of epidemiological data on the relationship between injecting “salts” and HIV risk behaviours. This is particularly relevant in settings where injection drug use accounts for a substantial proportion of the HIV burden, such as in Kyrgyzstan, a former Soviet Republic. This study assessed whether injecting “salts” is associated with sexual and injection‐related HIV risk behaviours among people who inject drugs in Kyrgyzstan.

**Methods:**

The Kyrgyzstan InterSectional Stigma Study is a cohort of people who inject drugs in Kyrgyzstan's capital of Bishkek and the surrounding rural administrative division of Chuy Oblast. We conducted a cross‐sectional analysis using survey data collected from cohort participants between July and November 2021, which included information on injection drug use (including “salts”) and HIV risk behaviours. To minimize confounding by measured covariates, we used inverse‐probability‐weighted logistic and Poisson regression models to estimate associations between recent “salt” injection and HIV risk behaviours.

**Results:**

Of 181 participants included in the analysis (80.7% men, 19.3% women), the mean age was 40.1 years (standard deviation [SD] = 8.8), and 22% (*n* = 39) reported that they had injected “salts” in the past 6 months. Among people who injected “salts,” 72% (*n* = 28) were men, and most were ethnically Russian 59% (*n* = 23), with a mean age of 34.6 (SD = 9.6). Injecting “salts” was significantly associated with a greater number of injections per day (adjusted relative risk [aRR] = 1.59, 95% confidence interval [CI] = 1.30−1.95) but lower odds of using syringe service programmes in the past 6 months (adjusted odds ratio [aOR] = 0.20, 95% CI = 0.12−0.32). Injecting “salts” was also significantly associated with lower odds of condomless sex in the past 6 months (aOR = 0.42, 95% CI = 0.24−0.76) and greater odds of having ever heard of pre‐exposure prophylaxis (aOR = 4.80, 95% CI = 2.61−8.83).

**Conclusions:**

(PWID) people who inject drugs who inject “salts” are a potentially emergent group with increased HIV acquisition risk in Kyrgyzstan. Targeted outreach bundled with comprehensive harm reduction and pre‐exposure prophylaxis services are needed to prevent transmission of HIV and other blood‐borne viruses.

## 1 INTRODUCTION

For more than three decades, injection drug use has fuelled the HIV epidemic in Eastern Europe and Central Asia (EECA) [1, 2]. Kyrgyzstan's HIV epidemic, like most EECA countries, is largely concentrated among people who inject drugs, who represent one‐third of the HIV burden [3, 4]. While HIV prevalence in the general population is low (0.2%), prevalence among people who inject drugs is 18% [4]. Of concern, there has been an increasing trend towards a generalized HIV epidemic. Specifically, in 2015, the majority of people with HIV were people who inject drugs (51%) [5]. Yet, by 2021, a majority of incident cases were acquired via sexual transmission, which may reflect underreported injection risk among sexual partners of men who inject drugs [6, 7]. In response, Kyrgyzstan implemented syringe service programmes and methadone maintenance treatment (MMT) in both community and based settings for people who inject drugs [8].

Emerging evidence indicates that regional drug markets are shifting away from opioids and towards new psychoactive substances such as synthetic cathinones, which are commonly referred to as “salts” or “bath salts” (hereafter referred to as “salts”) [9]. The quantity of “salts” seized in EECA countries increased substantially from 116 kg between 2005 and 2010 to 11,000 kg between 2015 and 2020 [10]. Changes in drug preference have important implications for HIV prevention, especially since MMT is less effective for people with opioid use disorder who use multiple substances, including amphetamine‐type stimulants and “salts” [11]. Stimulant use is associated with sexual risk‐taking, multiplying potential HIV transmission/acquisition risks [12]. Despite the availability of harm reduction services, stimulants such as “salts” can increase injection frequency and sexual risk behaviours and are associated with HIV outbreaks among people who inject drugs in high‐income countries with low HIV incidence [13, 14, 15, 16, 17].

Qualitative data from Kyrgyzstan indicate that “salts” can be obtained through online vendors and are preferred among young people who inject drugs due to their stimulant effects and among older people who inject drugs when heroin availability is low [9]. Given the paucity of epidemiological data on associations between “salt” injection and HIV risk behaviours in a region where injection drug use accounts for approximately 25% of new HIV cases, we aimed to evaluate if people who inject “salts” have elevated HIV (sexual and injection) risks compared to people who inject drugs other than “salts” in Kyrgyzstan [18].

## 2 METHODS

### 2.1 Study recruitment and enrolment procedures

We used data from the Kyrgyzstan InterSectional Stigma (KISS) cohort study. Recruitment and enrolment procedures have been described previously [19]. Eligible participants were: (1) 18 years of age or older; (2) injected drugs in the past 30 days with visual confirmation of injection marks; (3) willing to undergo rapid HIV testing; and (4) resided in either Bishkek or Chuy Oblast with no plans to relocate in the next 12 months. Participants were recruited through two non‐random purposive sampling approaches to capture a diverse sample of people who inject drugs through street‐based harm reduction outreach (April–May 2021, *n* = 204) followed by snowball sampling to reach participants not actively engaged in MMT or (SSP) syringe service program services (October–November 2021, *n* = 75). Due to funding and time restrictions, only participants recruited through outreach were eligible for a follow‐up survey. Approval was obtained from the Institutional Review Boards at the University of California, San Diego and GLObal Research Institute (GLORI) Foundation, Kyrgyzstan.

#### Measures

##### Exposure

Our exposure of interest was injecting “salts” in the past 6 months (yes, no). Based on research team observations, beginning 6th July 2021, we added questions regarding “salts” use to the baseline survey for the 75 participants recruited via snowball sampling and to the surveys of 173 (of 204) participants recruited via outreach who completed the follow‐up survey; of whom 64 participants reported no injection drug use since baseline and skipped the “salts” questions. Of the 184 participants who were asked this item, three (1.6%) were excluded because they responded “don't know” to injecting salts and the remaining 181 are included in the current analysis.

##### Outcomes

Our outcomes of interest included injection‐ and sexual‐related HIV transmission risk behaviours as well as HIV prevention service use/knowledge. Past 6‐month injection risk behaviour outcomes include injection with (i) a used needle, (ii) shared equipment, (iii) average number of injections per day and (iv) injection frequency (daily, more than once a week, once a week, 1−3 times per month). Past 6‐month sexual risk behaviour outcomes include past 6‐month (v) condomless sex, (vi) sex with multiple partners (≥1 times) and (vii) exchanging sex for money, drugs or other goods. HIV prevention service‐related outcomes include (viii) past 6‐month SSP services, (ix) lifetime MMT use; and among participants who were HIV seronegative or newly diagnosed at baseline, we determined if they (x) had met national HIV testing guidelines (at least one HIV test in the past 12 months) based on date of self‐reported last HIV test prior to baseline, and (xi) if they had ever heard of pre‐exposure prophylaxis (PrEP).

##### Covariates

Socio‐demographic covariates included age (in years; continuous), sex at birth (male or female), city of recruitment (Bishkek or Chuy Oblast), ethnicity (Russian or not Russian), education (< high school or ≥ high school), unstable housing (defined as sleeping in any place other than a house or apartment the participant or someone they knew owned or rented in the past 6 months), lifetime history of incarceration and baseline HIV status. Key drug use‐related covariates include self‐reported age at first injection (in years; continuous), past 6‐month injection of two or more drugs (polysubstance use) and past 6‐month non‐fatal overdose.

### 2.2 Statistical analysis

Descriptive statistics were calculated for continuous (means and standard deviation) and categorical measures (frequencies and proportions). We used inverse probability of treatment weights (IPTW) to balance potential confounding variables between participants who injected “salts” and participants who did not before comparing outcomes between these exposure groups. These methods addressed concerns about potential selection bias resulting from the different recruitment methodologies. Further, since our focus was solely on the association between salt use and HIV risk behaviours, using IPTW avoided presenting associations with nuisance variables. When modelling a rare outcome, weighting to control for confounding can facilitate model convergence and reduce bias due to overfitting compared to traditional covariate adjustment [20, 21, 22].

Calculating each participant's IPTW was a two‐step process. First, we used logistic regression to regress the exposure on the contemporaneously measured covariates listed above, then used the fitted exposure model to derive the estimated propensity score (i.e. probability of each participants’ reported exposure level conditional on their measured covariates). Second, we calculated each participant's IPTW as the inverse (or reciprocal) of their estimated propensity score. The application of these weights is intended to create a pseudo‐population in which potential confounders (i.e. the covariates included in the exposure model) are statistically independent of “salt” injection status [23]. We calculated standardized differences to determine if weighting improved the balance of potential confounders between treatment groups “salts” injection versus no “salts” injection in the past 6 months [24, 25]. Standardized differences of |>0.20| were taken to indicate a meaningful imbalance in the corresponding covariates between treatment groups.

Within the weighted sample, we estimated associations between “salts” injection on binary outcomes as odds ratios (OR) with 95% confidence interval (CI) using logistic regression and on count‐based outcomes as relative risk (RR) with 95% CI using Poisson regression. Confounders with standardized differences |>0.20| were added as covariates to the weighted regression models. To account for the familywise error rate in multiple comparisons, we applied the Holm−Bonferroni correction to all *p*‐values [26]. All analyses were conducted using SPSS (28.01.0) and R (4.2.1). *p*‐Values <0.05 were considered statistically significant.

## 3 RESULTS

Of 181 participants included in the analysis, 22% (*n* = 39) reported that they had injected “salts” in the past 6 months (Table 1). Among people who injected “salts,” 72% (*n* = 28) were men, and most were ethnically Russian 59% (*n* = 23), with a mean age of 34.6 (SD = 9.6). Overall, weighting helped balance covariates between exposure groups; however, ethnicity, HIV status and polysubstance use remained imbalanced between treatment groups post‐weighting (corresponding standardized differences |>0.20|).

**Table 1:** Sample characteristics among people who injected “salts” in the past 6 months before and after weighting

	Before weighting			After weighting		
Characteristic	People who injected “salts” (*n* = 39)	People who did not inject “salts” (*n* = 142)	Standardized difference	People who injected “salts” (*n* = 177)	People who did not inject “salts” (*n* = 177)	Standardized difference
**Socio‐demographics covariates**						
Age (in years), mean (SD)	34.6 (9.6)	41.6 (7.9)	−0.80	40.5 (9.1)	40.3 (8.6)	0.02
Sex at birth, *n* (%)						
*Male*	28 (71.8)	118 (83.1)	−0.27	155 (87.8)	145 (82.0)	0.16
*Female*	11 (28.2)	24 (16.9)	0.27	22 (12.2)	32 (18.0)	−0.16
City of recruitment, *n* (%)						
Bishkek	15 (38.5)	73 (51.4)	−0.26	77 (43.8)	85 (48.2)	−0.09
Chuy Oblast	24 (61.5)	69 (48.6)	0.26	99 (56.2)	92 (51.8)	0.09
Ethnicity, *n* (%)						
*Russian*	16 (41.0)	73 (51.4)	−0.21	67 (38.1)	91 (51.3)	−0.27
*Not Russian*	23 (59.0)	69 (48.6)	0.21	109 (61.9)	86 (48.7)	0.27
Education, *n* (%)						
*< High School*	13 (33.3)	45 (31.7)	0.03	65 (36.8)	60 (33.6)	0.07
*≥ High School*	26 (66.7)	97 (68.3)	−0.03	112 (63.2)	117 (66.4)	−0.07
Unstable housing in the past 6 months, *n* (%)	25 (64.1)	59 (41.5)	0.46	85 (48.2)	79 (44.9)	0.07
Ever been incarcerated, *n* (%)	19 (48.7)	100 (70.4)	−0.45	109 (61.9)	118 (67.0)	−0.11
Baseline HIV status^a^, *n* (%)						
*HIV negative*	36 (92.3)	105 (73.9)	0.51	119 (67.4)	137 (77.4)	−0.23
*HIV positive*	3 (7.7)	37 (26.1)	−0.51	58 (32.6)	40 (22.6)	0.23
**Drug use‐related covariates**						
Age at first injection (in years), mean (SD)	20.5 (4.2)	20.8 (5.7)	−0.06	21.3 (4.0)	20.7 (5.6)	0.12
Injected two or more drugs in the past 6 months	35 (89.7)	90 (63.4)	0.65	161 (91.0)	121 (68.2)	0.59
Overdose in the past 6 months	9 (23.1)	15 (10.6)	0.34	18 (10.4)	21 (11.6)	−0.04

Abbreviation: SD, standard deviation.

^a^ HIV test results assessed via OraQuick™ Rapid HIV antibody test.

In the weighted models and after adjustment for confounders (Table 2), injecting “salts” was significantly associated with a greater number of injections per day (adjusted RR [aRR] = 1.59, 95% CI = 1.30−1.95) and lower odds of having injected drugs with a used needle (adjusted OR [aOR] = 0.38, 95% CI = 0.18, 0.83). Injecting “salts” was associated with lower odds of engaging in past 6‐month condomless sex (aOR = 0.42, 95% CI = 0.24, 0.76), but greater odds of past 6‐month sex with multiple partners (aOR = 1.82, 95% CI = 1.05−3.15) and exchanging sex for resources (aOR = 4.16, 95% CI = 1.09−15.84). After adjusting for multiple comparisons, only the average number of injections per day and condomless sex retained statistical significance.

**Table 2:** Inverse‐probability‐of‐treatment weighted and covariate‐adjusted associations between injecting “salts” and HIV transmission risk behaviours and prevention service utilization

	Weighted and covariate adjusted			Holm−Bonferroni correction
	Estimated association	95% CI	*p* value	*p* value
**Past 6‐month injection risk behaviour outcomes**				
Injected with a used needle (yes. vs. no)	aOR = 0.38	0.18, 0.83	**.015**	**0.090**
Shared previously used injection equipment (yes vs. no)	aOR = 1.22	0.71, 2.11	.473	0.518
Average number of injections per day	aRR = 1.59	1.30, 1.95	**<.001** ^a^	**<0.01**
Frequency of injection drug use	aRR = 1.20	0.96, 1.47	.109	0.327
**Past 6‐month sexual risk behaviour outcomes**				
Any condomless sex (yes vs. no)	aOR = 0.42	0.24, 0.76	**.004** ^a^	**0.032**
Multiple (>1) sex partners (yes vs. no)	aOR = 1.82	1.05, 3.15	**.033**	**0.165**
Exchanged sex for money, drugs or other goods (yes vs. no)	aOR = 4.16	1.09, 15.84	**.037**	**0.165**
**HIV prevention service outcomes**				
Past 6‐month SSP use (yes vs. no)	aOR = 0.20	0.12, 0.32	**<.001** ^a^	**<0.01**
Lifetime MMT use (yes vs. no)	aOR = 1.29	0.83, 2.01	.259	0.518
Ever heard of PrEP (yes vs. no)	aOR = 4.80	2.61, 8.83	**<.001** ^a^	**<0.01**
Met HIV testing guidelines (yes vs. no)	aOR = 0.51	0.30, 0.86	**.011**	**0.077**

*Note*: Statistically significant differences are in **bold**.

Weights included age, sex at birth, city of recruitment, ethnicity, education, past 6‐month unstable housing, lifetime history of incarceration, baseline HIV status, age at first injection, past 6‐month injection of two or more drugs and past 6‐month non‐fatal overdose. Due to residual imbalances post‐weighting (i.e. standardized difference >.2), fitted weighted regression models additionally adjusted for the following variables as covariates: HIV status, ethnicity and past 6‐month injection of two or more drugs.

Abbreviations: aOR, adjusted odds ratio; aRR, adjusted relative risk; CI, confidence interval; IDU, injection drug use; MMT, methadone maintenance treatment; PrEP, pre‐exposure prophylaxis; SSP, syringe service programme.

^a^ Retained statistical significance after applying Holm−Bonferroni correction for multiple comparisons.

Participants who reported injecting “salts” had significantly lower odds of reporting past 6‐month SSP use (aOR = 0.20, 95% CI = 0.12−0.32) or having met HIV testing guidelines in the (aOR = 0.51, 95% CI = 0.30−0.86). Injecting “salts” was also associated with an increased odds of having heard of PrEP (aOR = 4.80, 95% CI = 2.61, 8.83). Statistical significance for HIV testing was not retained after adjusting for multiple comparisons.

## 4 DISCUSSION

Overall, we found significant differences that help to build an epidemiological profile of people who inject “salts” in Kyrgyzstan. This profile suggests that on average, people who injected “salts” were significantly younger, more likely to be recently unstably housed but less likely to have ever been incarcerated compared to people who did not inject “salts.” This underscores the potential for HIV transmission via injection and sexual risk behaviours in this population.

Despite injecting more frequently, participants who injected “salts” were significantly less likely to have accessed SSPs for sterile syringes in the past 6 months compared to those who did not inject “salts” suggesting the potential for increased concern for injection‐related transmission. Conversely, participants who injected “salts” were significantly less likely to report having condomless sex in the past 6 months and more likely to have heard of PrEP. While not significant in the corrected models, there was still an observed positive association with other elevated sexual risk behaviours, namely having multiple partners and exchanging sex for resources. This suggests some potential for elevated risk of sex‐related transmission in this group, and underscores the need to leverage PrEP awareness campaigns to facilitate increased screening for salt use and promote evidence‐based HIV prevention strategies in this population (e.g. SSP, HIV testing and PrEP linkage). These findings are consistent with qualitative data from Kyrgyzstan indicating that the short‐lived stimulant effects of “salts” require more frequent injecting and are associated with increased sexual risk [9].

In 2016, only two EECA countries, Tajikistan and Estonia, met the UNAIDS recommended target of providing at least 200 needles per year per person who injects drugs [1]. Previous modelling indicates that stimulant injection, including “salts,” represents a substantial proportion of new HIV acquisitions, thereby necessitating greater availability of high‐coverage SSPs [27]. However, lower engagement in SSPs suggests that current services and outreach targeting people who inject “salts” in the EECA are likely inadequate. Additional research is needed to understand how SSPs can more effectively reach people who inject “salts” and identify barriers to utilizing of those services. Surveillance of drug trends is needed to inform how harm reduction services can more rapidly adapt and respond to shifting drug markets and preferences.

Our study is subject to several limitations. First, inference based on findings from a small sample should be considered exploratory. Due to the cross‐sectional nature of the data, interpretation of these associations should not be considered causal. As data were collected from two sites in or near a large urban setting, findings should not be considered generalizable to all people who inject “salts” in Kyrgyzstan. Recruitment occurred during different phases of the COVID‐19 pandemic, and may have affected some individuals’ willingness to respond to our multi‐method recruitment strategy. Finally, both exposures and outcomes were based on self‐report, which could be imprecise or underreported due to recall or social desirability.

## 5 CONCLUSIONS

In conclusion, “salts” injection could be an emerging threat to the HIV response in the EECA region with implications to expand harm reduction services tailored specifically to the needs of this group who may be at increased risk for HIV acquisition.

## COMPETING INTERESTS

No competing interests to declare.

## AUTHORS’ CONTRIBUTIONS

RK wrote the first draft of the manuscript and conducted statistical analyses. ZB provided technical assistance with statistical analyses. DW and TLP critically reviewed and edited the manuscript. AK and NS collected data and critically reviewed the manuscript. JC conceptualized analysis, assisted with statistical analyses and drafted the manuscript. LRS provided technical assistance with statistical analyses, drafted the manuscript and critically edited the manuscript.

## FUNDING

This work was supported by the National Institutes of Health and Canadian Institutes of Health Research. Award Numbers R21TW011785, K01DA043421 and K01DA055521 from the National Institute on Drug Abuse and Fogarty International Center and CIHR PJH‐175382 from the Canadian Institutes of Health Research. The San Diego Center for AIDS Research, Health Equity Sociobehavioral Science Core provided expert consultation on study design and recruitment strategies (P30 AI036214).

## DISCLAIMER

The content is solely the responsibility of the authors and does not necessarily represent the views of the National Institutes of Health and Canadian Institutes of Health Research. Dan Werb is supported by the St. Michael's Hospital Foundation. Findings and their interpretation do not represent the views of the funder.

## Data Availability

The data that support the findings of this study are available from the PI Dr. Laramie Smith (laramie@ucsd.edu) upon reasonable request.
